# Retrieval of broken intra-medullary guide wire in femoral shaft fracture: A case report and surgical technique^☆^

**DOI:** 10.1016/j.tcr.2023.100865

**Published:** 2023-06-04

**Authors:** Kamparsh Thakur, Akshay Sharma, Manish Prasad, Imroz Jindal

**Affiliations:** a Dept of Orthopaedics, Military Hospital, Jammu, Jammu and Kashmir, India; b Dept of Orthopaedics, Military Hospital, Jaipur, Rajasthan, India; c Dept of Orthopaedics, Indian Field Hospital Level II plus, Juba, UNMISS, South Sudan; d Department of Orthopaedics, Armed Forces Medical College, Pune, India

**Keywords:** Broken guide wire, Intramedullary, Fracture shaft femur

## Abstract

Intramedullary Nailing is a standard procedure for fixation of various fractures in orthopedic practice. With the procedure also important is being aware of the complications and methods of managing them. A broken or stuck hardware can result in these procedures. We present a case with broken guide wire in a case of femoral shaft fracture in a young individual and the technique we used to retrieve it retrogradely from the fracture site. This technique shall be a useful technique to all the orthopedic surgeons if such a situation arises.

## Introduction

Broken Hardware removal is a nightmare during surgery for any orthopedic surgeon. There are multiple reported instances of broken instrument or implants which needed some expertise for removal [[1], [2], [3], [4], [5], [6]]. Hardware in soft tissue can be easily retrieved, however the scenario is different when it is embedded in the bone, especially if it is intramedullary. Intramedullary broken nails have been retrieved using various techniques by different authors [6,7]. We had a different situation while managing a patient with fracture shaft of femur, wherein the initial guide wire used for making entry point broke during reaming and had to retrieved from the fracture site. Given the shape and a hollow nature of intramedullary implant and options to hold it can be retrieved using different techniques, but a solid and small intramedullary implant retrieval can pose problems difficult to tackle while extracting it [3,4]. The location of the stuck hardware can eventually not only make the reaming troublesome, but also not allow the intramedullary implant to gain access of the canal to fix the fracture.

We present this case report showing an event of broken guide wire and its subsequent retrieval from the fracture site. The experience was a learning for the surgeon themselves and shall be helpful for any surgeon encountering a similar situation.

## Methods

A 23-year-old male had presented with comminuted fracture shaft of femur following a Road traffic accident. The trauma profile was normal with no associated injuries. The fracture was temporarily stabilized with skeletal traction. It was decided to fix the fracture with Intramedullary Interlocking (IMIL) Nail. He was taken for the surgery on the next day.

The patient was given Spinal Anesthesia and placed on the fracture table and 5 cm incision was given 5 cm proximal to greater trochanter (GT). The entry point was attempted using pointed guide wire, which was confirmed on the c-arm on AP and lateral views. As the guide wire was being over reamed with the entry reamer, the guide wire broke at the junction of the GT. Initial extraction was attempted using a blunt forceps but was unsuccessful. We decided to retrieve the wire using a hollow mill. However, during the extraction process, the guide wire got pushed further inside into the canal.

**Fig. 1:**
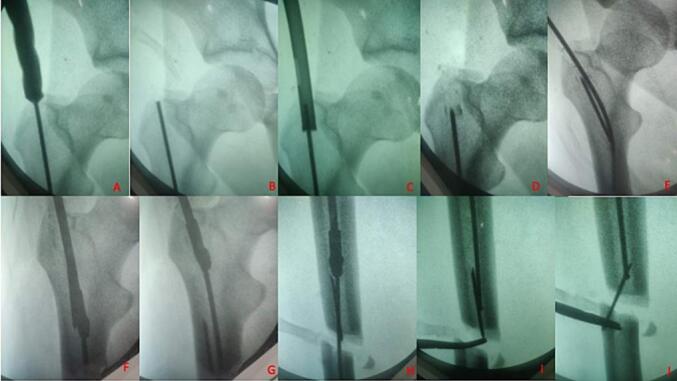
Sequential images of the c-arm showing the broken guide wire and its removal from the fracture site.

The location of the guide wire prevented us from proceeding ahead with next steps of surgery. After consultation with the senior surgeon, it was then decided to retrieve the wire from the fracture site. The entry made using the hollow mill allowed us to gain access of the canal using the nail guide wire. The canal was initially reamed with the smallest reamer (8 mm) just adjacent to the broken guide wire, to provide a path for the wire to push ahead into the fracture site. Subsequently a larger sized reamer was used to push the guide wire intramedullary into the fracture site. The guide wire once at the fracture mouth could be easily retrieved using a lateral incision and blunt forceps (Fig. 1).

Following retrieval of the guide wire the fracture was fixed using IMIL nail.

## Discussion

Broken hardware is an occurrence which an orthopedic surgeon must witness at some point of time during his or her practice, and to be prepared and aware of handling the situation is a necessity in today's world. The incidence of the same is reported in the literature with nailing process [8].

Broken hardware can be left inside the bone if not interfering with the fixation or at a risk of migration. In our case the broken guide wire was at imminent risk of interfering with fixation, as it would have not allowed the further reaming possible and desirable implant size usage, thus it was essential for us to retrieve it.

There are multiple reported cases of guide wire breaking in locations like femoral neck and their retrieval techniques [1,2]. These can be removed using pituitary forceps, over-reaming open procedures or arthroscopy assisted [2,5]. However, an intramedullary broken or stuck implant is a different case scenario which inadvertently requires its removal. A similar case with intertrochanteric fracture was encountered by Kasik et al. where they had a broken guide wire and were successful in retrieving it retrograde from the distal anterior femoral cortex after tapping the guide wire at proximal end. We in our case faced similar scenario but in a case of femoral shaft fracture and we too had to come up with a solution on the operating table and were successful in retrieving it.

The above technique is an additional way of retrieving the broken or stuck implant and will be helpful to all the operating surgeons should the situation arise.

## Conclusion

Broken instrument or implant are one of the most difficult situations to tackle in an orthopedic surgeon's practice. One should be aware of the methods to handle it. The present report shows one of the many solutions to this problem and shall be beneficial to every surgeon landing in a similar situation.

## Declaration of competing interest

None.
